# Active Glycogen Synthase in the Liver Prevents High-Fat Diet-Induced Glucose Intolerance, Decreases Food Intake, and Lowers Body Weight

**DOI:** 10.3390/ijms24032574

**Published:** 2023-01-29

**Authors:** Iliana López-Soldado, Joan J. Guinovart, Jordi Duran

**Affiliations:** 1 Institute for Research in Biomedicine (IRB Barcelona), The Barcelona Institute of Science and Technology, 08028 Barcelona, Spain; 2 Centro de Investigación Biomédica en Red de Diabetes y Enfermedades Metabólicas Asociadas (CIBERDEM), 28029 Madrid, Spain; 3 Department of Biochemistry and Molecular Biomedicine, University of Barcelona, 08028 Barcelona, Spain; 4 Institut Químic de Sarrià (IQS), Universitat Ramon Llull (URL), 08017 Barcelona, Spain; 5 Institute for Bioengineering of Catalonia (IBEC), The Barcelona Institute of Science and Technology, 08028 Barcelona, Spain

**Keywords:** glycogen, glycogen synthase, glucose, liver, food intake, high-fat diet, ATP

## Abstract

Many lines of evidence demonstrate a correlation between liver glycogen content and food intake. We previously demonstrated that mice overexpressing protein targeting to glycogen (PTG) specifically in the liver—which have increased glycogen content in this organ—are protected from high-fat diet (HFD)-induced obesity by reduced food intake. However, the use of PTG to increase liver glycogen implies certain limitations. PTG stimulates glycogen synthesis but also inhibits the enzyme responsible for glycogen degradation. Furthermore, as PTG is a regulatory subunit of protein phosphatase 1 (PP1), which regulates many cellular functions, its overexpression could have side effects beyond the regulation of glycogen metabolism. Therefore, it is necessary to determine whether the direct activation of glycogen synthesis, without affecting its degradation or other cellular functions, has the same effects. To this end, we generated mice overexpressing a non-inactivatable form of glycogen synthase (GS) specifically in the liver (9A-MGS^Alb^ mice). Control and 9a-MGS^Alb^ mice were fed a standard diet (SD) or HFD for 16 weeks. Glucose tolerance and feeding behavior were analyzed. 9A-MGS^Alb^ mice showed an increase in hepatic glycogen in fed and fasting conditions. When fed an HFD, these animals preserved their hepatic energy state, had a reduced food intake, and presented a lower body weight and fat mass than control animals, without changes in energy expenditure. Furthermore, 9A-MGS^Alb^ animals showed improved glucose tolerance when fed an SD or HFD. Moreover, liver triacylglycerol levels that were increased after HFD feeding were lower in these mice. These results confirm that increased liver glycogen stores contribute to decreased appetite and improve glucose tolerance in mice fed an HFD. On the basis of our findings, strategies to preserve hepatic glycogen stores emerge as potential treatments for obesity and hyperglycemia.

## 1. Introduction

Glucose is stored in the liver in the form of glycogen. The metabolism of this polysaccharide is controlled by the activities of two key enzymes, namely glycogen synthase (GS), which catalyzes the incorporation of glucose to a preexisting glycogen molecule, and glycogen phosphorylase (GP), which catalyzes the reverse reaction [1]. Both enzymes are regulated inversely by phosphorylation by several kinases: GS is inactivated by phosphorylation at multiple sites, whereas GP is activated by phosphorylation at a single site [2,3]. Conversely, the dephosphorylation of GS and GP by protein phosphatase 1 (PP1) leads to the activation of the former and the inactivation of the latter. Several PP1 regulatory subunits bind to glycogen and PP1, facilitating the interaction of the phosphatase with the glycogen-bound GS and GP, and thus promoting their dephosphorylation [4]. Among these regulatory subunits, protein targeting to glycogen (PTG, also called PPP1R3C or PPP1R5), which is expressed in many tissues, has been shown to regulate glycogen stores in several animal models [5,6,7]. In mammals, there are two GS isoforms, namely liver glycogen synthase (LGS), whose expression is specific to this organ, and muscle glycogen synthase (MGS), which is expressed in all other tissues [8].

We have previously shown that mice overexpressing PTG specifically in the liver, and which have increased glycogen content in this organ are protected from high-fat diet (HFD)-induced obesity by reduced food intake [9]. These animals maintain the hepatic energy state upon long-term fasting [10]. Furthermore, the overexpression of PTG in the liver ameliorates the diabetic and obesity phenotype in a db/db mouse model of obesity and diabetes as a result of a decrease in appetite [11]. These results allowed us to hypothesize that liver glycogen plays a key role in regulating food intake [9] and controls insulin sensitivity, gluconeogenesis, lipid metabolism, and ketogenesis upon nutrient deprivation [10]. However, the overexpression of PTG to increase liver glycogen implies certain limitations. As PTG is a regulatory subunit of PP1, a phosphatase that controls many cellular functions, the overexpression of PTG could impair PP1 function, leading to unpredictable effects beyond the regulation of glycogen metabolism. Furthermore, PTG not only induces the activation of GS, but also the inactivation of GP. 

In this context, it is necessary to determine whether the direct activation of glycogen synthesis, without affecting the degradation of this polysaccharide or its other cellular functions, has the same effects. To this end, we used our conditional mouse model overexpressing a non-inactivatable form of MGS (9A-MGS). Nine phosphorylation sites of 9A-MGS were mutated to alanine; therefore, this enzyme cannot be inactivated by phosphorylation [12]. The overexpression of 9A-MGS in several tissues induces a marked accumulation of glycogen [12,13,14,15]. We combined these mice with albumin Cre mice [16] to direct the expression of 9A-MGS specifically in the liver (9A-MGS^Alb^ mice).

Our results confirm the impact of liver glycogen on food intake and suggest that strategies to preserve hepatic glycogen stores may provide a treatment for obesity and hyperglycemia. 

## 2. Results

**Figure 1:**
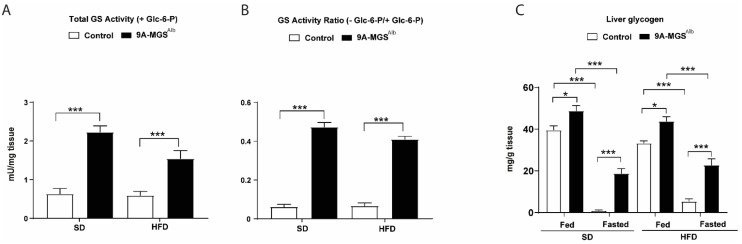
Characterization of 9A-MGS^Alb^ mice fed a standard diet (SD) or a high-fat diet (HFD). Control and 9A-MGS^Alb^ mice aged 6 weeks were fed an SD or HFD for 16 weeks. Fed and 16 h fasted mice were killed. (**A**) Liver Total GS activity under fed conditions. (**B**) Liver GS activity expressed as the ratio of (-) Glc-6-P/(+) Glc-6-P under fed conditions. (**C**) Liver glycogen content under fed conditions or a 16 h fast. Data are mean ± SEM. *n* = 6–10/group. * *p* < 0.05, *** *p* < 0.001.

Control and 9A-MGS^Alb^ mice were fed an SD or HFD. As expected, total GS activity (Figure 1A) and the GS activity ratio (Figure 1B) were higher in 9A-MGS^Alb^ compared to control mice. When fed either an SD or an HFD, 9A-MGS^Alb^ mice showed higher amounts of liver glycogen than control animals (Figure 1C). Similarly, after an overnight fast, 9A-MGS^Alb^ mice maintained higher liver glycogen content than control littermates under both diets (Figure 1C).

**Figure 2:**
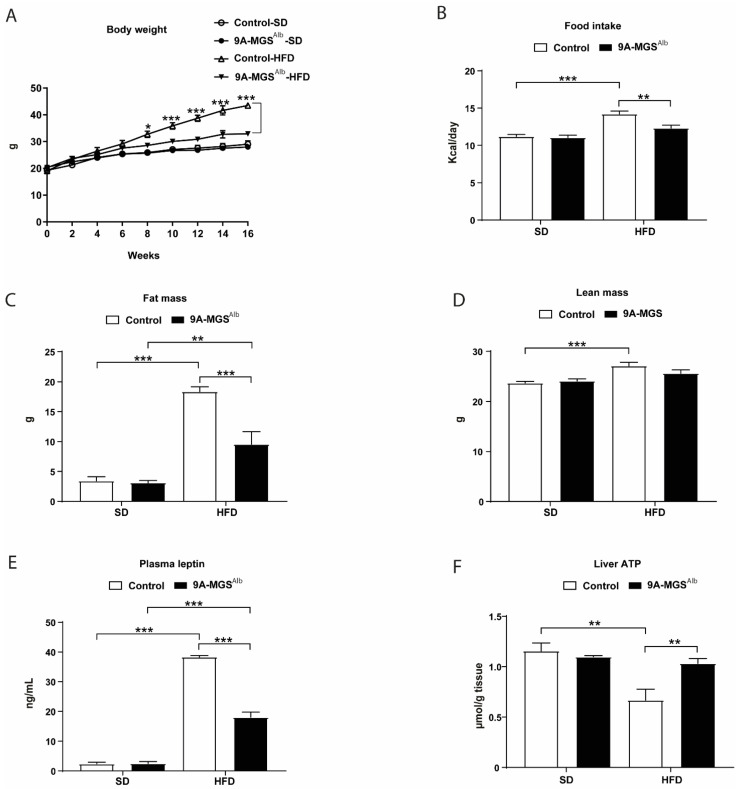
(**A**) Growth curve. Body weights were measured every other week. (**B**) Food intake. (**C**) Fat mass. (**D**) Lean mass. (**E**) Plasma leptin under fed condition. (**F**) Liver ATP under fed condition. Data are mean ± SEM. *n* = 6–10/group. * *p*<0.05, ** *p*<0.01, *** *p*<0.001.

Mice of both genotypes fed an SD had similar body weights (Figure 2A). Those on an HFD became obese (Figure 2A). However, the body weight increase in 9A-MGS^Alb^ animals fed an HFD was smaller than that registered in control mice under the same dietary conditions (Figure 2A). 9A-MGS^Alb^ mice on an HFD showed a reduced daily food intake compared with control mice on the same diet (Figure 2B). Under an HFD, 9A-MGS^Alb^ animals showed a less fat weight than control littermates (Figure 2C), while lean weight was similar in the two genotypes (Figure 2D). Consistent with this, serum leptin concentration was significantly lower in 9A-MGS^Alb^ mice fed an HFD compared to control mice on the same diet (Figure 2E). The ATP content of the livers of HFD-fed control mice was significantly reduced, while 9A-MGS^Alb^ mice on an HFD presented an ATP content similar to that of control mice on a standard diet (Figure 2F).

**Figure 3:**
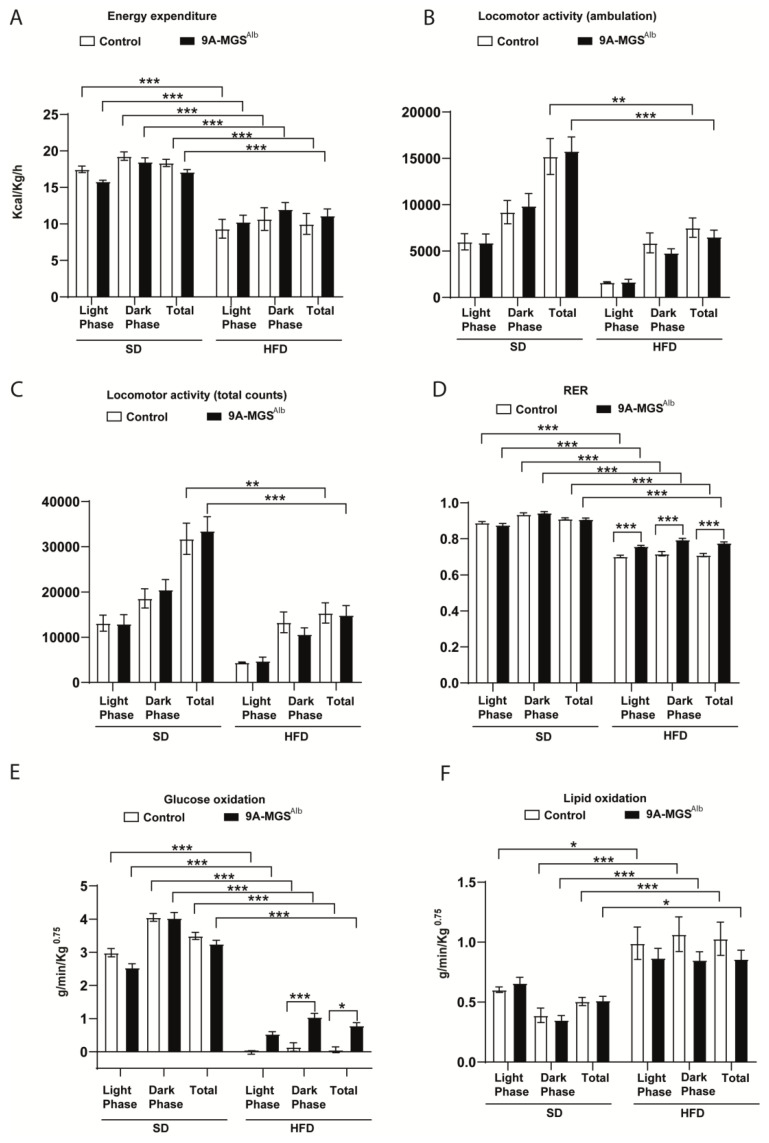
(**A**) Energy expenditure in control and 9A-MGS^Alb^ mice fed an SD and an HFD. (**B**) Locomotor activity (ambulation) in control and 9A-MGS^Alb^ animals fed an SD and an HFD. (**C**) Locomotor activity (total counts) in control and 9A-MGS^Alb^ mice fed an SD and an HFD. (**D**) Respiratory exchange ratio (RER) in control and 9A-MGS^Alb^ mice fed an SD and an HFD. (**E**) Glucose oxidation in control and 9A-MGS^Alb^ mice fed an SD and an HFD. (**F**) Lipid oxidation in control and 9A-MGS^Alb^ mice fed an SD and an HFD. Data are mean ± SEM. *n* = 4–7/group. * *p* < 0.05, ** *p* < 0.01, *** *p* < 0.001.

We also determined the daily energy expenditure (EE) during the dark and light phases in the two genotypes. When fed an SD or HFD, control and 9A-MGS^Alb^ mice had similar EEs (Figure 3A). No significant difference in locomotor activity was observed between the two genotypes (Figure 3B,C). In animals on the SD, there were no differences in the respiratory exchange ratio (RER) (Figure 3D). However, this parameter was increased in HFD-fed 9A-MGS^Alb^ mice, thereby indicating that these animals used more carbohydrates as an energy source than the control group (Figure 3D). These results were confirmed by calculating the amount of glucose oxidized, which was increased in 9A-MGS^Alb^ mice fed an HFD, especially during the dark phase (Figure 3E), while no differences were found in those on the SD (Figure 3E). However, 9A-MGS^Alb^ mice on an SD or HFD oxidized the same amount of lipids as control mice (Figure 3F). Animals on an HFD, independently of the genotype, showed a lower EE (Figure 3A) and lower glucose oxidation (Figure 3E) and higher lipid oxidation (Figure 3F) compared with animals on an SD.

**Figure 4:**
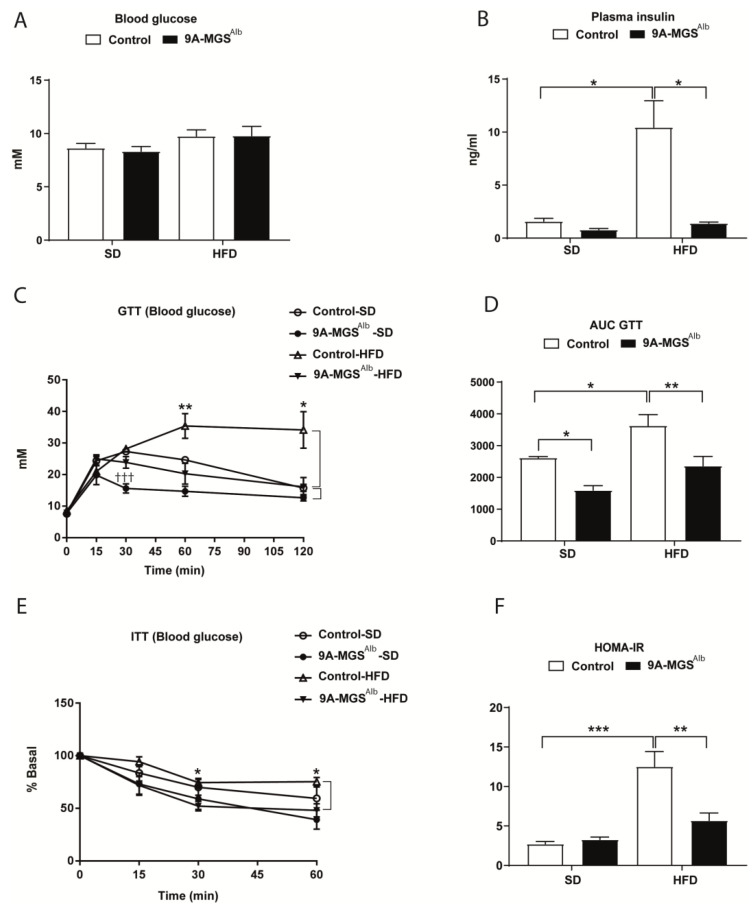
(**A**) Blood glucose concentration in fed condition. (**B**) Plasma insulin concentration in fed condition (* *p* < 0.05). (**C**) GTT curve for glucose. For GTTs, mice were fasted for 16 h and injected with 2 g glucose/kg body weight i.p. (††† *p* < 0.001, control-SD vs. 9A-MGS^Alb^-SD; * *p* < 0.05 and ** *p* < 0.01 control-HFD vs. 9A-MGS^Alb^-HFD). (**D**) Area under the curve (AUC) for GTT (* *p* < 0.05, ** *p* < 0.01). (**E**) ITT curve for glucose. For ITTs, mice were fasted for 6h and injected with 0.75 units insulin/kg body weight i.p. (* *p* < 0.05 control-HFD vs. 9A-MGS^Alb^-HFD). (**F**) HOMA-IR (** *p* < 0.01, *** *p* < 0.001). Data are mean ± SEM. *n* = 5–10/group.

9A-MGS^Alb^ animals had the same glucose levels as control animals regardless of the diet received (Figure 4A). However, the glucose tolerance test (GTT) indicated that 9A-MGS^Alb^ mice had better glucose tolerance, as reflected by a significant decrease in the area under the curve (AUC) compared with control mice, both in SD and HFD conditions (Figure 4C,D). Moreover, 9A-MGS^Alb^ mice showed lower levels of insulin compared with control mice on the same diet (Figure 4B). Accordingly, 9A-MGS^Alb^ mice on an SD or HFD presented improved insulin sensitivity compared to control littermates, as measured by an insulin tolerance test (ITT) (Figure 4E). Moreover, to determine relative insulin resistance (IR) across the groups, the homeostatic model assessment of insulin resistance (HOMA-IR) was calculated (Figure 4F). HFD feeding was important in increasing HOMA-IR in control animals, but not in 9A-MGS^Alb^ mice, suggesting that these animals were less IR than control mice.

**Figure 5:**
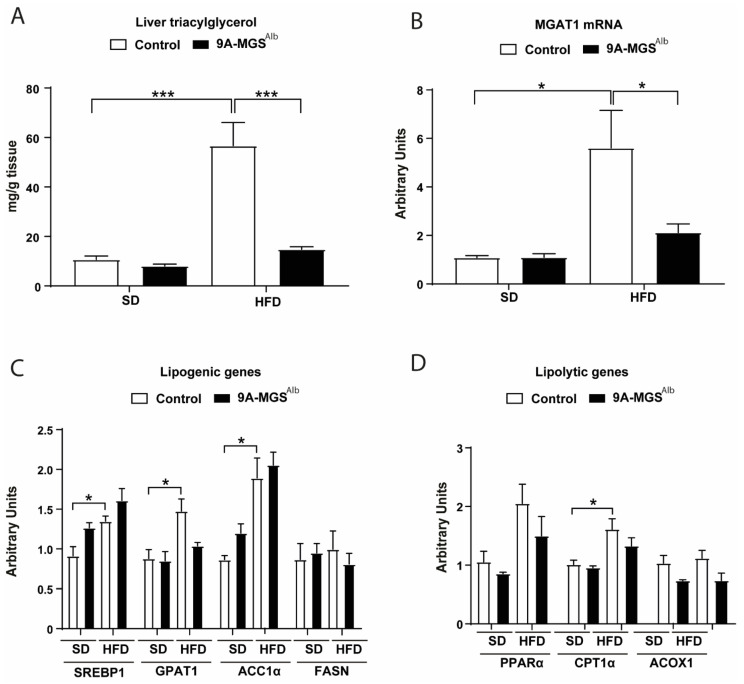
(**A**) Liver triacylglycerol in fed condition. (**B**) Quantitative real-time PCR showing relative mRNA levels of MGAT1 in the livers of mice fed an SD or HFD. (**C**) Quantitative real-time PCR showing relative mRNA levels of SREBP1, GPAT1, ACC1α, and FASN in the livers of mice fed an SD or HFD. (**D**) Quantitative real-time PCR showing relative mRNA levels of PPARα, CPT1α, and ACOX1. Data are mean ± SEM. *n* = 5–7/group. * *p* < 0.05, *** *p* < 0.001.

We next analyzed the storage of liver triacylglycerides (TAGs) in 9A-MGS^Alb^ animals. When fed an SD, these mice presented a similar liver TAG content as their control littermates under the same conditions. However, the increase in liver TAG induced by an HFD in control animals was prevented in 9A-MGS^Alb^ mice (Figure 5A). The lower hepatic TAG content in 9A-MGS^Alb^ mice fed an HFD was associated with a decrease in the expression of monoacylglycerol acyltransferase 1 (MGAT1) (Figure 5B), a microsomal enzyme that catalyzes the synthesis of diacylglycerol and TAG [17]. MGAT1 co-localizes to lipid droplets under conditions of enriching fatty acids, thereby contributing to TAG synthesis [18]. Moreover, the expression of lipogenic genes, such as sterol regulatory element binding protein 1 (SREBP1), glycerol-3-phophate acyltransferase 1 (GPAT1), and acetyl, CoA carboxylase (ACC1α), was upregulated in control mice fed an HFD, but not in 9A-MGS^Alb^ mice (Figure 5C). The expression of fatty acid synthase (FASN) was similar in all the groups (Figure 5C). To determine whether the decrease in hepatic steatosis was related to greater lipolysis, we measured several lipolytic-related genes in the liver. There were no differences in the expression of carnitine palmitoyltransferase 1α (CPT1α), peroxisome proliferator-activated receptor α (PPARα) or acyl-CoA oxidase (ACOX1) between the two genotypes under an SD or HFD (Figure 5D). 

## 3. Discussion

We previously showed that PTG overexpression in the liver results in a two-fold increase in hepatic glycogen in fed conditions [9], and in the maintenance of significant levels of the polysaccharide under fasting. To avoid the non-desired effects of PTG overexpression, here we generated 9A-MGS^Alb^ mice, which overexpress 9A-MGS specifically in the liver. The overexpression of 9A-MGS results in a dramatic increase in the amount of glycogen in tissues such as in the nervous system and skeletal muscle [12,13,14]. 

The increase in liver glycogen in 9A-MGS^Alb^ mice was accompanied by a decrease in food consumption and reduced weight gain under an HFD, as previously observed with PTG- overexpressing animals. These results confirm the impact of liver glycogen on food intake. The 9A-MGS^Alb^ mice presented higher levels of hepatic glycogen than control littermates in fed and fasting conditions, although the increase in glycogen during feeding was much smaller than in PTG-overexpressing mice. The most remarkable difference was observed in fasted animals, which maintained significant levels of glycogen. 

Control mice fed an HFD showed lower hepatic ATP. This observation is consistent with studies reporting that diabetes causes a decrease in ATP content in the liver [19,20]. The 9A-MGS^Alb^ mice maintained hepatic ATP content when fed an HFD. This finding thus reinforces our proposal that the maintenance of liver energy status contributes to decreased appetite and adiposity [9]. This effect is triggered by signals from the liver that are carried to the brain by vagal sensory neurons [21]. 

The HFD induced glucose intolerance and hyperinsulinemia, as previously described [9]. The 9A-MGS^Alb^ mice showed normal glucose tolerance and insulinemia, as previously shown for PTG-overexpressing animals [9]. It has been reported that pathological hyperinsulinemia drives diet-induced obesity (DIO) and its complications [22]. Therefore, the lower body weight observed in 9AMGS^Alb^ mice on an HFD could be due to the reversion of hyperinsulinemia. However, in Akita mice, a model with markedly reduced insulin production, we have demonstrated that increased liver glycogen leads to the long-term reduction of the diabetic phenotype, independently of circulating insulin [23]. 

Also of note was the effect of GS activation in decreasing the levels of hepatic TAGs after HFD feeding. It has been reported that the expression of genes encoding MGAT enzymes is induced in hepatic steatosis in humans [24], and that the MGAT1 pathway is critically important in the development of hepatic steatosis during DIO in mice [25]. Accordingly, MGAT1 levels were normalized in HFD-9AMGS^Alb^ mice. Several mechanisms could be involved in the reduction of intrahepatic fat observed in the HFD-9A-MGS^Alb^ mice, including decreased de novo lipogenesis and/or increased clearance through beta-oxidation [26]. However, no changes in lipogenesis or beta-oxidation were observed in the livers of these animals. Therefore, the decrease in TAG in these mice is probably a consequence of their lower food intake. 

In summary, 9A-MGS^Alb^ mice showed an increase in hepatic glycogen in fed and fasting conditions. When fed an HFD, these mice preserved their hepatic energy state, had a reduced food intake, and presented a lower body weight and fat mass compared to control animals. On the basis of our findings, the maintenance of glycogen levels, through a reduction in food intake, emerges as a promising strategy for the prevention of DIO, as well as for diet-induced glucose intolerance and insulin resistance. 

## 4. Materials and Methods

### 4.1. Mice

All procedures were approved by the Barcelona Science Park’s Animal Experimentation Committee and were carried out following the European Community Council Directive and the National Institute of Health guidelines for the care and use of laboratory animals. To obtain the expression of 9A-MGS specifically in the liver, the conditional 9A-MGS transgenic mouse model [12] was bred with an albumin promoter Cre recombinase-expressing animal (The Jackson Laboratory, Bar Harbor, ME, USA). Since the 9A-MGS expression cassette was introduced into the Hprt locus in the X chromosome, and to avoid mosaicism caused by female X chromosome inactivation, all the studies were conducted in male animals. All the mice studied were littermates. The animals were maintained on a 12:12 h light–dark cycle with free access to water and fed a standard diet (Harlan laboratories) or an HFD (45% kcal fat; catalog D12451 Research Diets, New Brunswick, NJ, USA) for 16 weeks, starting at 6 weeks of age. 

### 4.2. Body Mass Composition

Lean weight and fat weight were measured using magnetic resonance imaging (EchoMRI System, EchoMRI LLC, Houston, TX, USA). 

### 4.3. Metabolic Activity

Indirect calorimetry was performed using an eight-chamber Oxymax system (Columbus Instruments, Columbus, OH, USA) to measure energy expenditure, which was calculated from oxygen consumption and CO_2_ production. The mice were allowed to acclimate to the cages for 2 days before 3 cycles of 24 h measurements. EE (standardized for body weight), RER, glucose, and lipid oxidation were calculated as previously described [9]. Ambulatory and total locomotor activity was monitored by an infrared photocell beam interruption method.

### 4.4. Measurement of Cumulative Food Intake

To monitor food intake, the mice were housed individually and acclimatized for 1 week before the study. Food intake was measured daily for 5 consecutive days. 

### 4.5. Blood Parameters

Blood glucose levels were measured using a glucometer (Ascensia Breeze 2, Bayer HealthCare, Leverkusen, Germany). Blood was collected from the tail tip using microvette tubes with EDTA (Sarstedt, Nümbrecht, Germany). Plasma was collected after centrifugation at 3000× *g* for 20 min at 4 °C and kept at −80 °C until further analysis. Plasma insulin and leptin were analyzed by ELISA (Crystal Chem, Elk Grove Village, IL, USA). 

### 4.6. Tissue Preparation and Biochemical Analysis

When the animals were 6 months old, a cohort from each genotype and diet had their food removed at 4 p.m., and were sacrificed after a 16 h fast. A separate cohort of each genotype and diet was allowed access to food ad libitum and was sacrificed at 8 a.m. by cervical dislocation. The livers were quickly removed, placed in liquid nitrogen, and kept at −80 °C until further analysis. Glycogen was measured as previously described [27]. Briefly, frozen tissue was homogenized in 4 volumes of 30% KOH and heated at 100 °C for 15 min. Aliquots were then spotted on 31ET Whatman paper (Sigma-Aldrich, St. Louis, MO, USA). The papers were washed three times with 66% ethanol, which precipitates glycogen. The dried papers were incubated with amyloglycosidase (25 U/L Sigma-Aldrich, St. Louis, MO, USA) in 100 mM sodium acetate buffer at pH 4.8 to digest glycogen, and glucose release was determined by the reaction with hexokinase and glucose-6 phosphate dehydrogenase, following the original method described by Chan [28]. GS activity was determined as previously described in the presence or absence of Glc-6-P [29]. GS activity measured in the presence of saturating Glc-6-P [(+) Glc-6-P] corresponds to the total amount of enzyme, whereas measurement in its absence [(-) Glc-6-P] is an indication of the active (unphosphorylated) GS form. The (-) Glc-6-P/(+) Glc-6-P activity ratio is an estimation of the activity state of the enzyme. The intracellular concentration of ATP was measured in perchloric acid extracts by HPLC, as previously described [21]. Triacylglycerides (TAGs) in the liver were quantified in 3 mol/L KOH and 65% ethanol extracts following the method described by Salmon and Flatt [30], and using a kit (Sigma-Aldrich, St. Louis, MO, USA).

### 4.7. Glucose Tolerance, Insulin Tolerance Test and HOMA-IR

For GTTs, overnight-fasted (16 h) mice were injected with glucose 2 g/kg (body weight) i.p. Whole blood was drawn from the tail tip for glucose measurements. For ITTs, mice fasted for 6h were injected with insulin 0.75 units/kg (body weight) i.p., and glycemia was measured from tail blood taken at the indicated times after injection. HOMA-IR was calculated using the equation [(fasted glucose (mmol/L) x fasted insulin (µIU/mL)/22.5].

### 4.8. RNA Preparation and Quantitative RT-PCR

Liver RNA extraction, RT-PCR, and quantitative real-time PCR analysis were performed as described [31]. The following Taqman probe sets (Applied Biosystems, Waltham, MA, USA) were used for quantitative RT-PCR: MGAT1 (Mm00503358_m1); SREBP1 (Mm00550338_m1); GPAT1 (Mm00833328_m1); ACC1α (Mm01304257_m1); FASN (Mm00662319_m1); CPT1α (Mm01231183_m1); PPARα (Mm00440939_m1); and ACOX1 (Mm01246834_m1).

### 4.9. Statistical Analysis

Data are expressed as mean ± SEM. P values were calculated using two-way or one-way ANOVA with post hoc Tukey tests as appropriate.

## Abbreviations

HFD: high-fat diet; PTG, protein targeting to glycogen; PP1, protein phosphatase 1; SD, standard diet; GS, glycogen synthase; GP, glycogen phosphorylase; HOMA-IR, homeostatic model assessment of insulin resistance; LGS, liver glycogen synthase; MGS, muscle glycogen synthase; EE, energy expenditure; RER, respiratory exchange ratio; TAG, triacylglyceride; GTT, glucose tolerance test; ITT, insulin tolerance test; MGAT1, monoacylglycerol acyltransferase 1; SREBP1, sterol regulatory element binding protein 1; GPAT1, Glycerol-3-Phophate acyltransferase 1; ACC1α Acetyl, CoA carboxylase; FASN, fatty acid synthase; AUC, area under the curve; CPT1α, carnitine palmitoyltransferase 1α; PPARα, peroxisome proliferator-activated receptor α; ACOX1, acyl-CoA oxidase.
